# Better Local Disease Control With Mastectomy in Metaplastic Breast Carcinoma: Findings of a Retrospective Cohort

**DOI:** 10.7759/cureus.61517

**Published:** 2024-06-02

**Authors:** Nihan Turhan, Ecem Memişoğlu, Şermin Çoban Kökten, Nalan Turan Güzel, Elbrus Zarbaliyev

**Affiliations:** 1 General Surgery, Martyr Prof. Dr. İlhan Varank Sancaktepe Training and Research Hospital, Istanbul, TUR; 2 General Surgery, Kartal Dr. Lutfi Kırdar City Hospital, Istanbul, TUR; 3 Pathology, Kartal Dr. Lütfi Kırdar City Hospital, Istanbul, TUR; 4 Pathology, Martyr Prof. Dr. İlhan Varank Sancaktepe Training and Research Hospital, Istanbul, TUR; 5 General Surgery, Istanbul Yeni Yuzyıl University, Gaziosmanpasa Hospital, Istanbul, TUR

**Keywords:** breast cancer, surgical treatment, locoregional recurrence, triple-negative breast cancer, metaplastic breast cancer

## Abstract

Background: Metaplastic breast cancer (MBC) is a rare type of breast carcinoma with clinicopathological differences. The prognosis and treatment strategies for MBC are usually conflicting. In this study, we aim to present the clinicopathologic features, treatment strategies, and prognosis of our MBC patients.

Material and methods: In our retrospective study, 18 metaplastic breast cancer patients treated in our institution between January 2005 and December 2022 were evaluated. Demographic and clinicopathological characteristics, surgical and systemic treatment options, locoregional recurrences, distant metastases, and overall survival (OS) of the MBC patients were retrieved from the patient files.

Results: All patients were female; the median age was 54.42 ± 12.37 years. Most of the patients (n = 15, 83.33%) presented with palpable masses. Tumors were mostly triple-negative, with a high grade and a high Ki‑67 proliferation index. Spindle cell carcinoma and MBC with mesenchymal differentiation were the most common subtypes. Most of the patients underwent mastectomy (n = 11, 61.11%); breast-conserving surgery (BCS) was performed on seven (38,88%) patients. Lymph node positivity was detected in six of 18 patients (33.33%). Fewer patients (n = 4, 22.22%) received neoadjuvant chemotherapy. While local recurrence developed in two out of seven patients (28.57%) who underwent BCS, there was no local recurrence in patients who had mastectomy. The OS time varied according to tumor size and the presence of lymph node metastases (p <0.001; p = 0.005).

Conclusion: Metaplastic breast cancer is genetically heterogeneous and resistant to conventional treatment strategies. Mastectomy is still the surgical treatment method that is performed more frequently and provides better local control for patients with metaplastic breast cancer.

## Introduction

Metaplastic breast carcinoma (MBC) is a distinct pathological subtype of breast cancer and comprises 0.2%-5% of all breast tumors [1]. It was first described in 1973 by Huvos et al. and was formally recognized in 2000 [2, 3].

Metaplastic breast carcinoma most commonly exhibits triple-negative receptors via the absence of estrogen receptor (ER), progesterone receptor (PR), and human epidermal growth factor receptor 2 (HER2). Patients mostly present with a rapid growth of breast mass [4]. Even though MBC is similar to triple-negative breast cancer (TNBC) in terms of receptor status, most clinical studies show that MBC is more aggressive [1, 5-8]. Metaplastic breast carcinoma presents with less axillary nodal involvement and tends to spread hematogenously. Distant metastases are more common, especially in the brain and lungs. Treatment strategies are based on a small series of patient populations in the literature and are generally more aggressive for MBC [5, 9, 10]. Neoadjuvant chemotherapy is considered before surgery in stage II and III disease; however, disease control is not possible in most cases [11]. Disease-free survival (DFS) and overall survival (OS) are short [5, 10, 12].

Here, we reported 18 patients with MBC who were treated in our two institutions. The aim of this study is to present clinical and histopathological features of MBC disease, our treatment approaches, and results by comparing them with literature data.

## Materials and methods

This retrospective study was approved by the ethical committee of Martyr Prof. Dr. İlhan Varank Sancaktepe Training And Research Hospital in Istanbul, Turkey (approval number: 2022/156). We analyzed the medical charts of patients with breast cancer within an 18-year period (2005-2022). The following parameters were recorded: patient age, size of the tumor, ER, PR, HER2 receptor status, tumor grade, Ki-67 prognostic index, the WHO subgroup classification of metaplastic tumor, presence or absence of metastatic lymph node, germline mutations, surgical treatment modality, systemic treatment, radiation therapy, local recurrence or distant metastases, and patient OS. The final health status of the patients was verified by phone call. Patients whose treatment follow-up data could not be obtained were not included in the study.

All of the tumor specimens were reviewed by two pathologists. Immunohistochemical (IHC) staining for ER and PR was performed using a conventional detection method. Nuclear expression of ER and PR of more than 1% was considered positive [13]. Samples with 1% to 10% of cells staining ER positive were reported using a new reporting category, ER low positive [14]. Strong and complete membranous expression of HER2 in more than 10% of tumor cells was taken as positive HER2 expression on IHC. Fluorescence in situ hybridization (FISH) studies were performed for cases with equivocal HER2 [7]. The histological grade was determined according to the modified Bloom-Richardson classification.

Statistical analyses

Overall survival is defined as the length of time from the date of surgery to death from any cause or to the date of the last follow-up.

Categorical variables are expressed as n (%). Kaplan-Meier analysis was performed to investigate differences in overall survival time, and survival curves were compared using the log-rank test. Overall survival time is expressed as the mean ± standard error. If there are no ex-cases in the comparison groups, the overall survival time is expressed as minimum-maximum. The IBM SPSS Statistics software for Windows, Version 21.0 (IBM Corp., Armonk, NY: IBM Corp.) program was used for statistical analysis, and the type I error level was accepted as 5% in statistical analyses.

## Results

Of the 959 breast cancer patients treated in our institutions between January 2005 and December 2022, there were 20 (2.08%) MBC patients. Two patients with distant metastases at the time of initial admission were excluded from the study. The remaining 18 patients were included in the study. All patients were female. Germline analysis was performed on four patients, and no mutation was detected; therefore, it was not included in the statistical evaluation.

The clinical and histological features of the MBC patients are summarized in Table 1.

**Table 1:** The distribution of clinicopathological characteristics pT: pathological tumor stage; ER: estrogen receptor; PR: progesterone receptor; TNM: tumor, node, metastasis

Variables		Number of patients (n = 18)	Percentage (%)
Menopausal status	Premenopausal	6	(33.33%)
	Postmenopausal	12	(66.67%)
Side	Left	7	(38.89%)
	Right	11	(61.17%)
Clinical presentation	Mass	15	(83.33%)
	Radiological finding	3	(16.67%)
pT	T1	6	(33.33%)
	T2	7	(38.89%)
	T3	4	(22.22%)
	T4	1	(5.56%)
ER	Positive	4	(22.22%)
	Negative	14	(77.78%)
PR	Positive	2	(11.11%)
	Negative	16	(88.89%)
HER2	Positive	2	(11.11%)
	Negative	16	(88.89%)
Tumor grade	1	0	0
	2	2	(11.11%)
	3	16	(88.89%)
Ki-67 level	<20	2	(11.11%)
	≥20	14	(77.77%)
	Undetermined	2	(11.11%)
Lymph node status	N0	12	(66.67%)
	N1	6	(33.33%)
TNM stage	T1N0	6	(33.33%)
	T2N0	4	(22.22%)
	T2N1	3	(16.67%)
	T3N0	1	(5.56%)
	T3N1	2	(11.11%)
	T3N2	1	(5.56%)
	T4N2	1	(5.56%)
Histologic subtype	Mesenchymal differentiation	6	(33.33%)
	Spindle cell	7	(38.89%)
	Squamous	4	(22.22%)
	Mixed	1	(5.56%)

**Figure 1:**
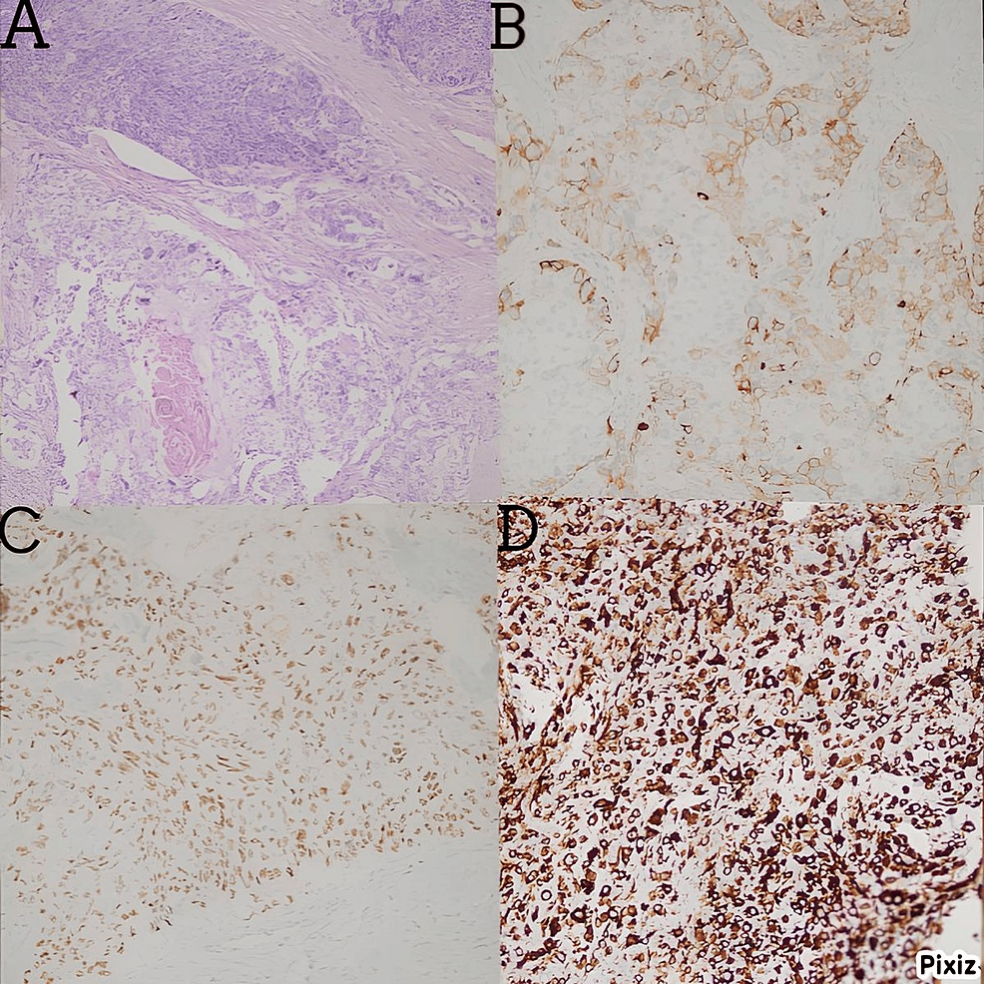
A) squamous cell metaplastic carcinoma (H&E x100); B) cytokeratin 5/6 positivity; C) P63 positivity; D) pan-cytokeratin positivity in spindle cell metaplastic breast carcinoma

The median age at diagnosis was 54.42 ± 12.37 years (range: 34-80 years). Most of the patients (n = 12, 66.67%) were postmenopausal. Three of the patients had a family history of breast cancer. Most of the patients (n = 15, 83.33%) presented with palpable masses. The mean tumor size was 3.6 cm (minimum: 2 cm; maximum: 8 cm). Spindle cell carcinoma and metaplastic carcinoma with mesenchymal differentiation were the most common subtypes. Most of the tumors were triple-negative (n = 14, 77.78%). Low levels of ER positivity (1%-10%) were seen in four patients. Two patients had HER2 positivity. Most of the tumors (n = 16, 88.89%) were grade 3. The Ki‑67 proliferation index was mostly high (≥ 20; n = 14, 77.77%). Examples of histopathological and immunohistopathological features are shown in Figures 1A-1D.

Systemic and surgical treatment features and outcomes are summarized in Tables 2-3. 

**Table 2:** Treatment strategies and outcomes MRM: modified radical mastectomy; BCS: breast conservative surgery; SLNB: sentinel lymph node biopsy; ALND: axillary lymph node dissection

Variables		Number of patients (n=18)	Percentage (%)
Surgical treatment	MRM	11	(61.11%)
	BCS+SLNB	6	(33.33%)
	BCS+ALND	1	(5.56%)
Radiotherapy	Yes	16	(88.88%)
	No	2	(11.11%)
Neoadjuvant chemotherapy	Yes	4	(22.22%)
	No	14	(77.77%)
Local recurrences	Yes	2	(11.11%)
	No	16	(88.88%)
Distant metastases	Lung	2	(11.11%)
	Bone	1	(5.56%)
	No	15	(83.33%)
Survival	Alive	14	(77.77%)
	Ex	4	(22.22%)

**Table 3:** Association between variables and overall survival Overall survival time is given as mean ± standard error. Since no ex-cases were observed in the groups indicated with *, the overall survival average could not be calculated, and the overall survival time is given as minimum-maximum. a: Log-rank test T: tumor stage; ER: estrogen receptor; PR: progesterone receptor; MRM: modified radical mastectomy; BCS+SLNB: breast-conserving surgery +sentinel lymph node biopsy; BCS+ALND: breast-conserving surgery +axillary lymph node dissection

Variables		Overall survival (month)	p-value^a^
Menopausal status	Premenopausal	67.83±10.19	0.981
	Postmenopausal	125.70±23.48	
Clinical presentation	Mass	120±76.28	0.415
	Radiological finding*	22-43	
T	T1 and T2	150±21.21	0.001
	T3 and T4	17.50±4.56	
ER	Positive*	18-108	0.171
	Negative	120±75.83	
PR	Positive*	18-18	0.521
	Negative	120±76.09	
HER2	Positive*	12-12	0.566
	Negative	120±76.03	
Tumor grade	2*	26-26	0.521
	3	120±76.09	
Kİ-67	<20	92±34.29	0.291
	≥20	155.16±16.24	
Lymph node status	N0	150±21.21	0.005
	N1	14±1.36	
Histological subtype	Mesenchymal differentiation*	3-36	0.214
	Others	120±77.01	
Surgical treatment	MRM	120±0	
	BCS+SLNB*	26-79	0.402
	BCS+ALND	120±75.83	
Radiotherapy	Yes	147.58±16.86	0.855
	No	120±0	
Neoadjuvant chemotherapy	Yes	28.67±5.99	0.493
	No	131.33±22.11	

Modified radical mastectomy was performed in 11 (61.11%) patients with American Joint Committee on Cancer (AJCC) stages II and III. Early-stage patients with small tumor diameters were selected for breast-conserving surgery (BCS). Breast-conserving surgery was performed on seven (38.88%) patients. Axillary lymph node dissection (ALND) was performed in 12 patients, and a sentinel lymph node biopsy (SNLB) was done in six patients. In the end, six patients (33.33%) had axillary lymph node metastasis.

Treatment strategy was determined for each patient individually in multidisciplinary oncology meetings. All of the patients received both systemic and surgical treatment. In total, four patients received neoadjuvant chemotherapy. No pathological complete response (pCR) was achieved in any of the patients who received neoadjuvant chemotherapy. A partial pathological response was achieved in all patients. Half of the patients who received neoadjuvant chemotherapy underwent mastectomy, and the other half underwent BCS.

**Figure 2:**
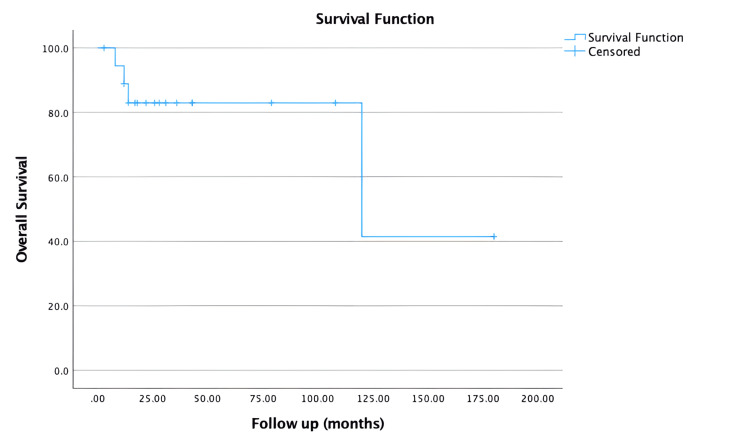
Overall survival curve according to the follow-up time

The main chemotherapy regimen was anthracycline and alkylating agents, followed by taxanes. Trastuzumab was administered to two HER2-positive patients. Patients with low ER positivity were given anti-hormonal therapy. Most of the patients (n = 16, 84.21%) received radiation therapy. Two patients (28.57%) who underwent BCS presented with locoregional recurrence in the remaining breast tissue on the same side. While no local recurrence was observed in any of the patients who underwent mastectomy, a case of new breast cancer development was detected in the other breast in one of the mastectomized patients. Systemic metastases occurred in three (15.78%) patients (lung metastases in two patients and bone metastases in one patient) in the first five-year period following the operation. Overall survival in months is shown in Figure 2. 

Four patients (21.05%) had died. The median survival time was found to be 27 (minimum: eight; maximum: 180) months. The five-year survival rate of the patients included in the study was determined to be 82.96%, and the 10-year survival rate was 41.48%. Overall survival time varied according to tumor size. The survival times of patients in the T3-T4 group were shorter compared to the T1-T2 group (p≤0.001).

Also, it was determined that OS time varied depending on the presence of lymph node metastasis. The survival time of N1 patients was shorter than that of N0 patients (p = 0.005).

## Discussion

Metaplastic breast carcinoma is a rare breast cancer subtype. The term metaplasia defines the non-glandular change of cancer cells from pluripotent stem cells as epithelial, mesenchymal, or both. The morphologic types of tumor cells determine the histologic classification of MBC [3]. The WHO classification system of phenotypes of metaplastic breast carcinoma includes low-grade adenosquamous carcinoma, fibromatosis-like metaplastic carcinoma, squamous cell carcinoma, spindle cell carcinoma, metaplastic carcinoma with mesenchymal differentiation, myoepithelial carcinoma, and mixed metaplastic carcinoma [15].

In previous studies, MBC was mostly seen in women over 50 years of age and in the postmenopausal stage [10, 16]. Corso et al. presented that the majority of MBC was a triple‐negative subtype (88.7%), with grade 3 (95.3%), pN0 (70.6%), and high levels of Ki‐67 (93.5%) [10]. Similarly, Ong et al. presented MBC patients with higher proportions of ER−, PR−, and HER2− biomarker statuses, a higher clinical and pathologic stage, and less frequent nodal involvement. In their study, 70.3% of MBC patients had TNBC [9].

In our study, the majority of MBCs affected postmenopausal women. Tumors showed a triple-negative phenotype, presented with high grade (G3), and had high Ki‐67 levels. The clinicopathological characteristics of our patients with MBC are based on the literature.

According to the literature, mastectomy was performed more commonly in MBC patients, even though BCS was regarded as a suitable option [9, 10, 17-19]. Presentation at an advanced stage, typically with larger tumors, is one of the main reasons to opt for mastectomy [4, 10, 19]. By the way, local recurrences appear to be common in BCS. Elalfy et al. stated high recurrence rates of up to 28.3% (17 out of 60 patients) during a relatively short follow-up (median 15 months) period [20]. Tzanninis et al. also reported a high local recurrence risk after BCS. They emphasized the need for a negative surgical margin of at least 3 cm wide and adjuvant radiotherapy to prevent local recurrence in patients undergoing BCS [21]. In addition to this, failure to achieve the desired results in reducing the primary mass in patients given neoadjuvant chemotherapy leads them away from BCS. For triple-negative breast carcinoma, the pCR rate for neoadjuvant chemotherapy is as high as 30%-50% [22]. However, it has been reported in the literature that pCR rates in MBC patients are quite low [20, 23-25]. Haque et al. experienced a 9.8% (88 out of 903) pCR response in MBC patients [25]. They presented that the majority of patients with MBC undergoing pCR still underwent mastectomy (62.5%). Wong et al. also observed a poor response to neoadjuvant chemotherapy, with only one patient out of 44 patients achieving pCR, 22% of the patients showing no clinical or radiological response, and 27% having progression [11]. Similarly, Tadros et al. reported progression in 40% and no response in 30% to neoadjuvant chemotherapy in MBC patients [26].

In our institutions, in accordance with the literature, mastectomy was the preferred surgical method, especially for high-grade tumors. It is important to emphasize that the local recurrence rate was low in patients who underwent mastectomy in our series.

Receiving neoadjuvant chemotherapy did not affect the choice of our surgical method. In our opinion, the decision to give neoadjuvant chemotherapy to patients with MBC should be evaluated in multidisciplinary tumor councils.

Racka et al. analyzed prognostic factors in 450 patients with MBC; 70% of the patients had axillary clearance and 30% had SNLB. Thirty percent showed metastatic nodes. They stated that the lymph node stage, lymphovascular invasion, and histologic subtype determined the outcome [27]. In another study done by Ong et al., 2,500 patients were evaluated retrospectively for metaplastic breast cancer treatment and outcome. They showed that MBC patients were more likely to receive ALND (35.2% vs. 32.2%, all p≤0.001) compared to non-MBC patients. However, MBC patients more frequently had negative lymph nodes than non-MBC patients (20.0% vs. 10.6%, p<0.001), which concluded that ALND may be overutilized in MBC [9]. Song et al. reported that MBC was usually associated with less axillary nodal involvement, but the risk of developing distant metastases was greater than in typical breast adenocarcinoma. So, it was believed that hematogenous spread was important for MBC [5, 12]. 

In our patient group, ALND was used more liberally, and metastasis rates were found to be low, similar to the literature. However, it is noteworthy that the prognosis was worse for patients with axillary lymph node metastases in our study.

Mao et al. showed that prognosis in MBC patients was associated with the patient's age, tumor grade, TNM stage, and surgical treatment [28]. Papatheodoridi et al. stated that the advanced TNM stage was associated with an increased risk of death [17]. Similarly, Hu et al. presented that, tumor size and lymph node status were risk factors for survival [29]. In our study, the advanced pT stage was associated with worse overall survival.

Given that MBC is a rare disease, information on the prognosis of histopathological subtypes is also scant. Spindle cell carcinoma, squamous cell carcinoma, and carcinoma with mesenchymal differentiation were reported as aggressive subtypes [27]. However, in Corso et al.’s series, they observed a worse prognosis for MBCs associated with mixed components [10]. Özkurt et al. found that the mesenchymal subtype had the worst survival outcome as well [1]. In the end, there is no definite subtype of MBC with a worse prognosis presented in the literature. In our study, we found no relationship between MBC subtypes and prognosis.

Although most of the patients have triple-negative tumors, some MBC variants appear to overexpress the HER2/neu oncoprotein [30]. In the study conducted by Lei et al. on 58 MBC patients, HER2 positivity was associated with a high risk of recurrence and a poor prognosis. Targeted therapy was recommended for these patients [31]. Gao et al. showed in their study that HER2-positive MBC patients can achieve long-term progression-free survival and complete remission if they receive anti-HER2 targeted therapy [32]. Invasive breast cancers are considered ER-positive if at least 1% of the cancer nuclei stain is positive, and these patients are considered candidates for endocrine therapy [13]. However, new guidelines mention that low-level (1%-10%) ER expression is heterogeneous in both behavior and biology. They have genetic expression profiles very similar to ER-negative cancers. They are considered eligible for anti-hormone treatment, but the benefits of anti-hormone therapies for these patients are limited [14, 33]. In our patient series, individualized anti-hormonal and targeted treatments were given according to the patients' receptor positivity status.

It has been shown that the prognosis is worse in MBC patients compared to invasive adenocarcinomas and triple-negative tumors of the breast. In Aydiner et al.'s study, 17.1% of patients died and 25.5% had disease progression at a median follow-up of 28 months. Lymph nodes, lungs, liver, and brain were major metastatic sites involved in disease progression [34]. Similarly, Ong et al. found a five-year OS of 72.7% for MBC patients [9]. In another study by Corso et al., the median OS for MBC patients was 5.0 years (range 2.3-8.8), and the three-year rate of OS was 86.1% [10]. Gortman et al. found a five-year disease-free survival of 57.1% and a five-year OS of 57.1% [5]. The OS rate in our patients with MBC worsened with long follow-up, consistent with the literature.

Patients with MBC are generally resistant to conventional chemotherapy agents, and more efficient treatment regimens are required. Metaplastic breast cancers are genetically heterogeneous and harbor somatic mutations, most frequently in TP53, PIK3CA, and PTEN. Also, they have been associated with overexpression of PD-L1 and tumor-infiltrating lymphocytes. The IHC for expression of high molecular weight cytokeratins and basal markers such as CK14, CK5/CK6, e‑cadherin, AE1/AE3, 34betaE12, and EGFR are considered to be important prognostic markers [5]. For treatment with immunotherapeutic agents, germline tests and detailed IHC and molecular testing of their tumors are recommended for MBC patients [35]. We could not evaluate the results of those parameters because the number of patients who underwent these tests was insufficient in our patient group.

Our study has some limitations, including a small cohort of patients, insufficient germline tests, a small number of detailed IHC analyses, a relatively short follow-up, and the retrospective nature of the study.

## Conclusions

Although surgery, chemotherapy, and radiotherapy are routinely used in treatment, outcomes for MBC are still poor. As increased tumor size and lymph node positivity were associated with poor prognosis, early diagnosis is very important for a better prognosis. We think that mastectomy is a more suitable surgical option for local control of the disease. Further research is important for understanding the mechanisms of carcinogenesis, identifying clinically relevant prognostic factors, and determining the most appropriate treatment options for this disease.
